# Industry and Occupation in the Electronic Health Record: An Investigation of the National Institute for Occupational Safety and Health Industry and Occupation Computerized Coding System

**DOI:** 10.2196/medinform.4839

**Published:** 2016-02-15

**Authors:** Matthew Schmitz, Linda Forst

**Affiliations:** ^1^ Environmental and Occupational Health Sciences School of Public Health University of Illinois at Chicago Chicago, IL United States

**Keywords:** medical informatics, occupation code, industry code, NIOCCS, occupational health, occupation+electronic health record

## Abstract

**Background:**

Inclusion of information about a patient’s work, industry, and occupation, in the electronic health record (EHR) could facilitate occupational health surveillance, better health outcomes, prevention activities, and identification of workers’ compensation cases. The US National Institute for Occupational Safety and Health (NIOSH) has developed an autocoding system for “industry” and “occupation” based on 1990 Bureau of Census codes; its effectiveness requires evaluation in conjunction with promoting the mandatory addition of these variables to the EHR.

**Objective:**

The objective of the study was to evaluate the intercoder reliability of NIOSH’s Industry and Occupation Computerized Coding System (NIOCCS) when applied to data collected in a community survey conducted under the Affordable Care Act; to determine the proportion of records that are autocoded using NIOCCS.

**Methods:**

Standard Occupational Classification (SOC) codes are used by several federal agencies in databases that capture demographic, employment, and health information to harmonize variables related to work activities among these data sources. There are 359 industry and occupation responses that were hand coded by 2 investigators, who came to a consensus on every code. The same variables were autocoded using NIOCCS at the high and moderate criteria level.

**Results:**

Kappa was .84 for agreement between hand coders and between the hand coder consensus code versus NIOCCS high confidence level codes for the first 2 digits of the SOC code. For 4 digits, NIOCCS coding versus investigator coding ranged from kappa=.56 to .70. In this study, NIOCCS was able to achieve production rates (ie, to autocode) 31%-36% of entered variables at the “high confidence” level and 49%-58% at the “medium confidence” level. Autocoding (production) rates are somewhat lower than those reported by NIOSH. Agreement between manually coded and autocoded data are “substantial” at the 2-digit level, but only “fair” to “good” at the 4-digit level.

**Conclusions:**

This work serves as a baseline for performance of NIOCCS by investigators in the field. Further field testing will clarify NIOCCS effectiveness in terms of ability to assign codes and coding accuracy and will clarify its value as inclusion of these occupational variables in the EHR is promoted.

## Introduction

The links between work and health have been long understood [1]. Illnesses and injuries that arise out of employment are amenable to primary, secondary, and tertiary prevention, but prevention requires identification of hazards as well as knowledge of associated health conditions. Inclusion of information about employment in medical records could aid physicians and other health professionals in the recognition of work-related illnesses and injuries. Such information could also highlight working conditions that interfere with general health and well-being and stymie treatment efforts. Furthermore, capture of employment variables would allow for intervention research and surveillance of work related patterns of illness and injury at the population level [2-4]. After earlier attempts at automated coding of work variables for governmental agencies in the United States [5], the development and widespread use of electronic health records (EHRs) presents a unique opportunity to advance the collection and incorporation of information on industry and occupation (I&O) into patients’ medical records [6].

In its 2010 report, “Reducing Environmental Cancer Risk - What We Can Do Now”, the President’s Cancer Panel recommended routine assessment of occupational history and the incorporation of such information into the medical record in order to better assess potential workplace exposures and related risk of chronic illnesses such as cancer [7]. The National Prevention Council, headed by the US Surgeon General, also called for the inclusion of occupational and environmental risk assessment in the patient medical history in its 2011 National Prevention Strategy [8]. An Institute of Medicine 2011 report, “Incorporating Occupational Information in Electronic Health Records”, concluded that incorporation of occupational information in EHRs could contribute to improving individual and population health care and issued a number of recommendations to the US National Institute for Occupational Safety and Health (NIOSH) and its partners to guide the process; these include adopting Standard Occupational Classification (SOC) coding standards for use in EHRs, assessing the feasibility of autocoding occupational health information, and assessing the impact on meaningful-use goals of incorporating occupational information into EHRs, specific objectives that must be met to qualify for Centers for Medicare & Medicaid Services Incentive Programs meant to ensure that implementation of EHRs improves clinical outcomes and population health [9]. As a result of these recommendations, NIOSH developed the National Industry and Occupation Computerized Coding System (NIOCCS), and following on to earlier work in this area, a Web-based system that translates “industry” and “occupation” text into standardized codes. The NIOCCS system has had limited evaluation, to date.

The goal of this investigation was to evaluate the quality of I&O coding of data obtained in a community-based, health care needs assessment. Specific objectives were to: (1) determine interrater reliability of hand coding of “industry” and “occupation” variables using the 2010 Standard Occupational Codes; (2) determine the ability of NIOCCS to assign codes to “industry” and “occupation” responses from the general public (potential patients); and (3) to evaluate the relationship between hand-coded versus NIOCCS-assigned SOC codes.

## Methods

### University of Illinois Survey on Neighborhood Health

In 2013-2014, the University of Illinois Survey on Neighborhood Health (UNISON) was conducted, as this health care system’s community health needs assessment required by the Patient Accountability and Affordable Care Act (ACA) [10]. A sampling scheme was constructed for a study of 1400 individuals, approximately half of them reached through a door-to-door survey according to a randomized, block design; the other half that were surveyed were current patients with specific chronic diseases.

A survey tool was created from existing national surveys to collect patient-reported information about health behaviors, health care access and utilization, prevalence of disease conditions, quality of life indicators, and knowledge of the ACA. Basic biometric screening was done and those who answered the survey were invited to come to university clinics for laboratory testing. There were 3 questions that were inserted by investigators to use for the current study: (1) current employment status; (2) type of business or industry working in (respondents could select from a list or write in a response); and (3) job title (write-in, only). For every currently employed individual, responses to questions 2 and 3 were downloaded to a MS Excel spreadsheet and used in this analysis. No identifying information was obtained.

### Coding

The 2010 SOC system is used to classify workers into occupational categories for the purpose of collecting, calculating, or disseminating data [11]. This system has been adopted by several federal agencies in order to harmonize collection of work-related variables in a variety of demographic, employment, and health oriented databases. The 2010 version classifies workers into one of 840 detailed occupations according to their definitions. To facilitate classification, detailed occupations are combined to form 461 broad occupations, 97 minor groups, and 23 major groups. Detailed occupations in the SOC with similar job duties, and in some cases skills, education, and training, are grouped together. The coding scheme gives 2 digits for the 23 major categories, followed by a hyphen, and 4 more digits for more finely described categories (XX-XXXX). For example, a home health aide would be coded Healthcare Support Occupations (31-0000) for major group, (31-1000) for minor group, Nursing, Psychiatric, and Home Health Aides (31-1010) for broad occupation, and Home Health Aide (31-1011) for detailed occupation [11].

The investigators obtained the 2010 SOC system of codes and individually hand-coded the data down to the most detailed level of classification possible based on the information provided by each respondent [11]. They then met and came to a consensus if their codes differed.

**Figure 1:**
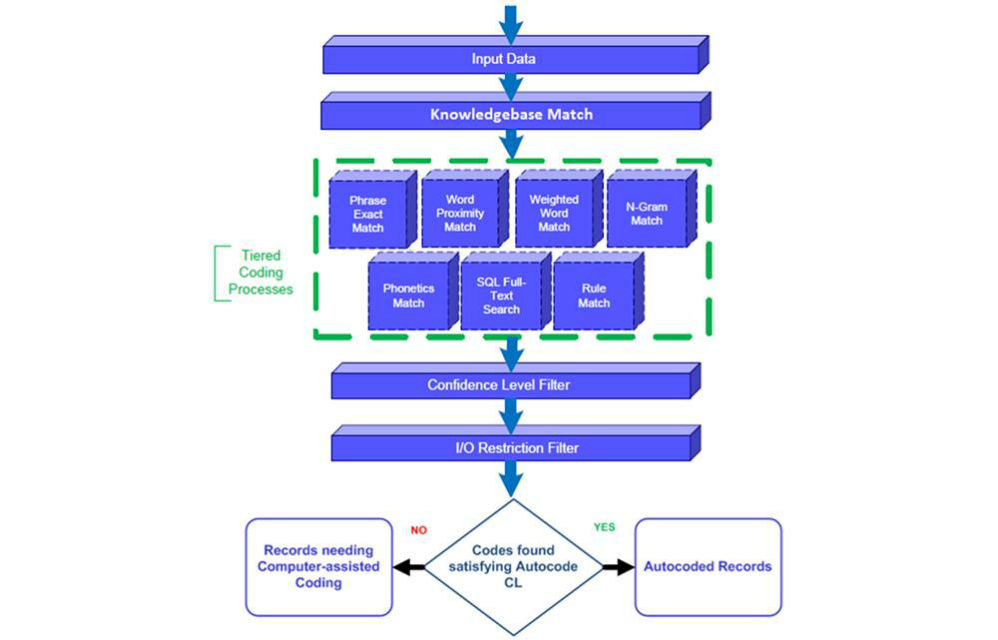
The US National Institute for Occupational Safety and Health (NIOSH) Industry & Occupation Computerized Coding System (NIOCCS) Coding Engine [12]. I&O: industry and occupation.

The NIOSH Industry & Occupation Computerized Coding System (NIOCCS) Version 2.0 was used to autocode the data according to 2010 SOC codes, separately from the hand coding. NIOCCS is a Web-based system that translates text into standardized I&O codes [12]. It was designed for use by researchers, government agencies, state health departments, and other organizations that collect or evaluate information using I&O. Its purpose is to provide a tool that reduces the high cost of manually coding I&O information, while simultaneously improving uniformity of the codes. The NIOCCS Coding Engine design has processes that cover phrase-based and word-based, exact match and proximity match, and weighted and not-weighted matching. Each process has its specialty of best-fit coding areas to enhance the combined coding ability. Figure 1 shows the NIOCCS coding scheme [12].

The dataset of survey participants’ responses on employment was used for NIOCCS autocoding for the first 2 digits (industry) and the last 4 digits (occupation) using both the high confidence level (90% cutoff) and medium confidence level (70% cutoff) criteria. Records processed using the high confidence threshold require that NIOCCS has 90% or greater confidence of accuracy for matching, whereas the medium confidence threshold only requires 70% or greater confidence of accuracy for coding to occur. The degree of confidence is based on the degree of fidelity of the variable to the actual code, with the “high” confidence level requiring stronger probability of a match (less “fuzziness”) than “medium” confidence level allows. We used either the industry data (first 2 digits) selected by the respondent from a list, or the write-in response when “other” was selected. The occupation data, the last 4 digits, were written in by the respondent (not selected off a list).

### Data Analysis

The kappa statistic was used to assess the reliability of manual versus NIOCCS autocoding. The first 2 digits were compared between hand and electronic coding, as were the last 4 digits. The proportion of records containing I&O information that are autocoded out of all the records submitted for coding is called the “production rate”. The industries and occupations that could not be coded by NIOCCS resulted in unbalanced contingency tables, a row by column table where the manually assigned codes for each respondent constitute the rows and the NIOCCS-assigned codes constitute the columns, for both the 2-digit and 4-digit comparisons. A contingency table is one where rows and columns are set up to analyze for associations between the two. In order to obtain correct kappa statistics, an approach suggested by Crewson [13] was used to create balanced contingency tables through the creation of dummy observations covering all possible rating scenarios in a separate stratum from the original data. Using this approach, SAS PROC FREQ calculates the kappa statistics separately for the strata containing the original data and the dummy observations. All 6 digits were not evaluated because of the extremely high number of potential rating categories and practical limits of computing power. SAS v.9.4 (SAS Inc, North Carolina) was used for this analysis.

## Results

### Response Breakdown From Door-to-Door Survey

Responses were obtained from 740 individuals in the door-to-door survey, with 359 (48.5%) currently working. The breakdown of “industry” based on hand coding, considered the gold standard for I&O coding, is shown in Table 1. There were 6 of the 23 industries that comprised over 50% of the responses.

**Table 1:** Industrial sectors of 359 working individuals surveyed in the UNISON study.

Industry	Number	%	Cum %
Office and administrative support (43-XXXX)	37	10.3	10.3
Management (11-XXXX)	32	8.9	19.2
Health care support (31-XXXX)	30	8.4	27.6
Food preparation and serving (35-XXXX)	29	8.1	35.7
Education, training, and library (25-XXXX)	28	7.8	43.5
Personal care and service (39-XXXX)	24	6.7	50.2
Transportation (53-XXXX)	23	6.4	56.6
Sales and related (41-XXXX)	20	5.6	62.2
Business and financial operations (13-XXXX)	18	5.0	67.2
Production (51-XXXX)	18	5.0	72.2
Protective service (33-XXXX)	14	3.9	76.1
Health care practitioners and technical (29-XXXX)	13	3.6	79.7
Arts, design, entertainment, sports, and media (27-XXXX)	11	3.1	82.8
Building and grounds cleaning and maintenance (37-XXXX)	11	3.1	85.9
Life, physical, and social science (19-XXXX)	9	2.5	88.4
Computer and mathematical (15-XXXX)	8	2.2	90.6
Community and social service (21-XXXX)	8	2.2	92.8
Construction and extraction (47-XXXX)	7	1.9	94.8
Job title “None”, left blank, or unknown	6	1.7	96.5
Legal (23-XXXX)	5	1.4	97.9
Installation, maintenance, and repair (49-XXXX)	4	1.1	99.0
Architecture and engineering (17-XXXX)	2	0.6	99.6
Farming, fishing, and forestry (45-XXXX)	1	0.3	99.9
Temp worker	1	0.3	100.0

### Production Rates for “High” and “Medium” Confidence Levels

Table 2 shows the production rates (the percentage autocoded) for “high” and “medium” confidence levels. For the high confidence algorithm, the production rates of “industry” (the first 2 digits) range from 115/359 (32.0%) to 129/359 (35.9%), with the “write-ins” performing a little better (23/65, 35% autocoded) than those selected off a card (94/294, 31.9% autocoded). Production rates were higher for the medium confidence level (176/294, 59.8% to 38/65, 58%). Similar results were obtained for the production rates of “occupation” (the last 4 digits) at both confidence levels.

**Table 2:** Production rates of autocoding of I&O by 2- and 4- digit Standardized Occupational Codes obtained from 359 respondents in a community survey, with industry selected from a list versus industry write-in.

	High confidence, %	Medium confidence, %
2-digit SOC (XX-XXXX), industry selected off card	32	49
2- digit SOC (XX-XXXX), industry write-in	36	58
4-digit SOC (XX-XXXX), industry selected off card	31	49
4-digit SOC (XX-XXXX), industry write-in	36	58

### Comparison of Manual Coding by Two Investigators

Both investigators manually coded all 359 responses. For the comparison of manual coding by each of the investigators, the 2-digit blinded coding yielded a kappa of 0.84 (95% CI 0.79-0.88) using 338 observations (21 missing); at the 4-digit level, a kappa of 0.58 (95% CI 0.52-0.63) using 337 observations (22 missing) was achieved. Investigators subsequently reached agreement at the 6-digit level (2 and 4, combined) on every case and used these agreed-upon codes for comparisons to the NIOCCS-generated codes. Hand coding versus NIOCCS coding yielded high kappa statistics at the 2-digit level, but low kappa statistics at the 4-digit level. The NIOCCS high confidence algorithm produced higher accuracy than the medium confidence level, but coded fewer observations. There was no difference in selection of industry off a card versus writing it in free form. All comparisons are shown in Table 3.

**Table 3:** Interrater reliability measures of SOC 2010 coding of I&O data by different coding methods.

Coding technique	n (missing)	Kappa (95% CI)
Investigator 1 x Investigator 2: 2 digit	338 (21)	0.84^a^ (0.79-0.88)
Investigator 1 x Investigator 2: 4 digit	337 (22)	0.58 (0.52-0.63)
Investigator-agreed x NIOCCS high: 2 digit	115 (244)	0.84^a^ (0.77-0.91)
Investigator-agreed x NIOCCS high: 2 digit (with write-in)	129 (230)	0.84^a^ (0.78-0.91)
Investigator-agreed x NIOCCS high: 4 digit	112 (247)	0.70 (0.62-0.79)
Investigator-agreed x NIOCCS high: 4 digit (with write-in)	129 (230)	0.68 (0.60-0.76)
Investigator-agreed x NIOCCS med: 2 digit	177 (182)	0.71 (0.65-0.78)
Investigator-agreed x NIOCCS med: 2 digit (with write-in)	207 (152)	0.71 (0.64-0.77)
Investigator-agreed x NIOCCS med: 4 digit	177 (182)	0.60 (0.52-0.67)
Investigator-agreed x NIOCCS med: 4 digit (with write-in)	207 (152)	0.56 (0.49-0.63)

^a^ agreement = high

## Discussion

### Relationship Between Work and Health

There is a growing appreciation of the relationship between work and health. First, “work” is a determinant of health in that job activities expose working people to illness and injury risks: workers who are exposed to chemical, biological, physical, ergonomic, and psychosocial hazards are more likely to experience adverse health effects associated with those hazards. Furthermore, health is affected by employment status, whether an individual is employed, unemployed, partially employed, or stably employed has an impact on health and well-being [14]. Second, employment in the United States is integrally related to health insurance coverage for both general health and work-related injury/illness, and health care coverage determines whether an individual has access to care or whether optimal health outcomes can be achieved as a result of a clinical encounter. Third, the workplace is increasingly being utilized as a venue for health promotion activities. Wellness programs are being implemented to prevent and control chronic health conditions based on the belief that a healthy workforce is a more productive workforce and will cost the employer less money due to absenteeism, presenteeism (low productivity while at work), and health insurance costs [15-17]. Finally, there are recent studies examining the transfer of workers’ compensation costs to general health insurance, to federal programs, to community health centers as nonreimbursable costs, and to indigent worker-patients, themselves [18-20]. Inclusion of information on I&O in the EHR is integral to providing information that can be utilized in health care encounters to improve the health and well-being of adult patients. On a population level, variables collected in the EHR can be used to study relationships between sociodemographics, hazardous exposure conditions, and health outcomes. The use of health records by state programs for surveillance of occupational illness and injury has increased in the last decade as a way to enhance case capture, target preventive efforts, and conduct research [21].

General guidelines for the interpretation of the kappa statistic have been suggested by Landis and Koch [22] as values <.00 indicating poor agreement, .00-.20 as slight, .21-.40 as fair, .41-.60 as moderate, .61-.80 as substantial, and .81-1.00 as almost perfect agreement. Fleiss [23] has also suggested guidelines characterizing kappa values below .40 as poor, .40-.75 as fair to good, and over .75 as excellent. While both sets of guidelines are arbitrary, they provide a general framework for the interpretation of kappa values. It is also important to remember that the magnitude of kappa values can be influenced by the prevalence of given attributes being rated, in this case the prevalence of employment in various industries and occupations among the sample population, as well as potentially by bias [24].

### Principal Results

Using the Landis and Koch [22] framework for interpreting the kappa statistic, the between-investigator agreement for 2-digit manual coding was “almost perfect”, as was the agreement between the manually coded classification and the NIOCCS high confidence model, while the agreement between the manually coded classification and the NIOCCS medium confidence model was slightly lower, but still “substantial”. The between-investigator agreement for 4-digit coding was moderate, the agreement between the manually coded classification and the NIOCCS high confidence model was substantial, and the agreement between the manually coded classification and the NIOCCS medium confidence model was moderate. Including respondent-provided write-in information for industry led to a slight increase in the number of observations autocoded by NIOCCS for both high and medium confidence models, but did not alter the kappa statistic for 2-digit coding and slightly decreased the kappa statistic for 4-digit coding as compared to the manually coded classifications.

If the investigator-agreed-upon manually coded classifications are taken as the true I&O classifications for this study, then the NIOCCS models performed near, but slightly below the accuracy performance goals of 10% or less error rate and 25% or less error rate for the high and medium confidence models respectively, when examining the 2-digit coding; however, it performed well below the performance goals for the 4-digit coding [25]. Importantly, the NIOCCS high confidence model achieved a comparable level of agreement for the 2-digit coding and a greater level of agreement for the 4-digit coding than was originally achieved between blinded investigators. However, a significant limitation of the model that may influence the overall agreement between manual and autocoded results is the production rate of the NIOCCS autocoding (ie, most of the entries do not get coded by NIOCCS and therefore cannot be compared with manual coding). Notably, there does not appear to be any pattern in regards to industry or occupation in the data that was not autocoded.

The goal of NIOCCS autocoding is to simplify and mechanize insertion of I&O variables into the EHR; production rate and accuracy are critical to streamlining inclusion of these variables. This study used a field-based survey to explore public use of NIOCCS for coding I&O. Data provided in Table 4 show that NIOCCS did not perform as well in this study as it has in other settings. Death certificates yield the highest production rates and coding by NIOSH performs better than it did for external investigators in this study.

**Table 4:** NIOCCS autocoding production rates (proportion autocoded) for this investigation versus other studies.

Data type		Year 2013, %	Year 2014 (Jan-Sept), %	Illinois ACA survey, %
**NIOCCS internal user (NIOSH personnel) results**				
	Death certificates	60	61	
	Surveys	34 MESA [25] & REGARDS [26] Survey Data	37 BRFSS [27] Survey Data	
**NIOCCS external user (non-NIOSH personnel) results**				
	Death certificates	64 [28]	64 [28]	
	Cancer registries	35 [28]	60 [28]	
	Surveys	49 [28]	50 [28]	32-36
	Other	52 [28]	57 [28]	
Average—all data types		51	55	
BRFSS-10 states [27]		31-55 (avg=42%)^a^		

^a^ Personal communication, NIOSH

### Production Rates of the National Industry and Occupation Computerized Coding System

The production rates of the NIOCCS high confidence model were 32% and 36% for the selected and write-in augmented data, respectively; the production rates using the medium confidence model were 49% and 58% for the selected and write-in augmented data. The observed production rates are below the predicted benchmark rate of around 50% for the high confidence and 60%-75% for the medium confidence model when coding survey data [25]. This may be indicative of the quality of the data provided by UNISON survey respondents rather than the performance of the NIOCCS autocoding, however, data acquisition from patients at home versus health care settings should not vary on that basis alone. Because of this, the findings of this study may indicate the need to develop better methods, training, or emphasis on the collection of more detailed information on I&O to support successful autocoding.

### Limitations

Because the same 4-digit occupational code can exist within different 2-digit industry code groups, there is a potential for misinterpreting 4 digit codes from different 2-digit major categories as an exact match, however, it is unlikely that this occurred at a frequency high enough to bias the results: the 2-digit codes are quite different, and if it had happened at a frequency high enough to have biased the results, then the 4-digit agreement between hand coding and NIOCCS would have been inflated compared to the 2-digit agreement, which they were not; they were essentially equivalent.

It should be noted that true reliability of the codes—both in the collection phase and in the coding phase—would best be evaluated by further questioning of respondents (patients) to assure that the given I&O are accurate descriptors of their actual industrial sectors and job titles. This would require a study that entails responses to the I&O questions, followed by more extensive questioning of the respondent. In addition, the ability to evaluate hazardous workplace exposure, consider risk, or promote health in the workplace would require more detailed questioning of the worker by the health care provider.

### Conclusions

This study provides important field testing for the NIOCCS system on data collected through an ACA-required community needs assessment carried out by a university health system; development of an autocoding system was recommended by the Institute of Medicine’s Committee on Occupational Information and Electronic Health Records. Our results showed that the NIOCCS accuracy performed near its expected benchmark levels for 2-Major Groups SOC coding (“industry”), but well below the expected benchmark for the 4-digit detailed occupation SOC coding level (“occupation”) [23]. In this study, NIOCCS production rates fell below the anticipated production rates for survey data. This study could serve as an important baseline performance measure for NIOCCS as NIOSH continually improves the system to specifically target autocoding and accuracy rates.

According to the UNISON group, “Information learned from UNISON Health will help us to understand the health needs of the diverse community served by the UI Health and to use this information to improve health care for those who need it the most” [10]. “Work” is an important, though often ignored, determinant of health. Knowledge of adult patients’ places of employment—both I&O—can serve to inform interventions to improve health and well-being on a population level. Utilization of the NIOCCS tool could aid both researchers and this particular health system in understanding the occupational makeup of the population within its service area by reducing the time and cost associated with manually coding I&O information. It could also aid in establishing uniformity of I&O codes contained within patients’ EHRs that could be used to inform physicians of a patient’s unique work history and risks of work related health conditions.

## Abbreviations

ACA: Affordable Care Act
EHRs: electronic health records
I&O: industry and occupation
NIOCCS: National Industry and Occupation Computerized Coding System
NIOSH: US National Institute for Occupational Safety and Health
SOC: Standard Occupational Classification
UNISON: University of Illinois Survey on Neighborhood Health
